# Movement guidelines for young children: Engaging stakeholders to design dissemination strategies in the Hong Kong early childhood education context

**DOI:** 10.3389/fpubh.2022.1007209

**Published:** 2022-11-29

**Authors:** Catherine M. Capio, Rachel A. Jones, Catalina Sau Man Ng, Cindy H. P. Sit, Kevin Kien Hoa Chung

**Affiliations:** ^1^Early Childhood Education Department, Education University of Hong Kong, Tai Po, Hong Kong SAR, China; ^2^Centre for Psychosocial Health, Education University of Hong Kong, Tai Po, Hong Kong SAR, China; ^3^Health Science Department, Ateneo de Manila University, Quezon City, Philippines; ^4^School of Education, University of Wollongong, Wollongong, NSW, Australia; ^5^Department of Sports Science and Physical Education, The Chinese University of Hong Kong, Tai Po, Hong Kong SAR, China

**Keywords:** physical activity, sedentary behavior, sleep, early childhood education, movement guidelines, dissemination

## Abstract

**Background:**

Early childhood is a critical period during which patterns of movement behaviors are formed. The World Health Organization had endorsed guidelines for physical activity, sedentary behavior and sleep over a 24-h time period, which had been adopted by the Center for Health Protection of Hong Kong. This paper reports on stakeholder engagements that were conducted to inform the design of strategies to disseminate the guidelines in early childhood education (ECE) settings.

**Methods:**

Using a mixed-methods study design, we sought to (a) assess the stakeholders' levels of awareness and knowledge of the Hong Kong movement guidelines for young children and (b) identify the factors that influence the uptake of the said guidelines. We conducted an online survey of early childhood education teachers (*N* =314), twelve focus groups involving teachers (*N* = 18) and parents (*N* = 18), and individual interviews of key informants (*N* = 7) and domestic workers who provide care for preschool-aged children (*N* = 7). Descriptive statistics were used for the quantitative data, and thematic analysis was performed on the qualitative data using an inductive and semantic approach following a realist framework.

**Findings:**

Our findings show that teachers were aware of the movement guidelines for young children, but their knowledge of the specific guidelines was deficient; parents and domestic workers had limited awareness and knowledge of the guidelines. Uptake of the movement guidelines is enabled by parent engagement, activities in the ECE centers, home-school cooperation, and community activities for children. The challenges include the time poverty of parents, local curriculum requirements, limited physical spaces, social values, and pandemic-related restrictions.

**Conclusion:**

We recommend that dissemination strategies in the ECE context should deliver knowledge content and support stakeholders in mitigating the challenges associated with time, space, and social conditions.

## Introduction

Early childhood is widely considered a critical period during which skills and patterns of health behaviors are formed, which are believed to track through childhood, adolescence and adulthood (1). In late childhood and adolescence, children tend to have reduced physical activity and increased sedentary time (2, 3). In Hong Kong, the government reports that <30% of children in kindergartens (i.e., 3–5 years old) accrue 180 min of physical activity per day. At the same time, the median time spent on sedentary screen time is 60 min (4). A longitudinal study showed that while 50% of primary school children (i.e., 6–10 years old) met the recommended 60 min of moderate to vigorous physical activity per day, this declined to 22% 2 years later (5). It appears that strategies to promote physical activity need to start in the early years. In the long term, physical activity not only contributes to preventing obesity but also upholds the children's right to active play (6).

The World Health Organization (WHO) released guidelines on physical activity, sedentary behavior and sleep – collectively known as movement behaviors – for children aged 5 years and younger in 2019 (7). The guidelines were developed to respond to the childhood obesity epidemic and to initiate surveillance and monitoring of children's movement behaviors over time (8). The WHO recognized that movement behaviors along a whole-day continuum, not only physical activity, contribute to the physical health and wellbeing of children (9). In earlier work, 24-h movement guidelines were launched in Canada, Australia, and South Africa, which encompassed physical activity, sedentary behavior, and sleep (10–12). Movement behaviors were considered to interact with each other, and their combined effects were larger than their distinct contributions (13). These earlier guidelines informed the WHO recommendations.

The Center for Health Protection (CHP) in Hong Kong adopted the guidelines by the WHO in 2020 but modified them to apply to children aged 2–6 years, which is the age of children attending early childhood education (ECE) centers (14). ECE in Hong Kong consists of pre-nursery, nursery, junior kindergarten, and senior kindergarten levels. It is not mandatory for children to be enrolled in ECE, but it has been reported that as of 2019, virtually all children attend ECE classes (15). The CHP specified recommendations for two age groups: (a) children aged 2 years or pre-nursery pupils and (b) children aged 3–6 years or nursery, junior kindergarten, and senior kindergarten pupils. There are variations in the recommendations for physical activity and sleep, but those for sedentary behavior are similar for the two age groups (see Table 1 for details). These movement guidelines have been disseminated through ECE centers that are participating in a wider public health campaign on young children's nutrition and physical activity. The movement guidelines are published as a “Physical Activity Guide” for ECE centers (14). It includes the rationale behind the guidelines and focuses on establishing a physical activity policy in the ECE centers.

**Table 1:** Recommendations for young children in Hong Kong on physical activity, sedentary behavior, and sleep (14).

**Age**	**Recommendations**
2 years old	• At least 180 min in a variety of *physical activities* of different intensity levels (including moderate to vigorous intensity physical activity) • No more than 1 h at a time when restrained in prams/strollers, high chairs, or seats; no more than 1 h of sedentary screen time per day (e.g., watching TV or using a computer, tablet computer or smartphone) • A range of 11–14 h of good quality sleep, including naps, with regular sleep and wake-up times
3–6 years old	• At least 180 min in a variety of *physical activities* of different intensity levels, including at least 60 min of moderate to vigorous intensity physical activity • No more than 1 h at a time when restrained in prams/strollers, high chairs, or seats; no more than 1 h of sedentary screen time per day (e.g., watching TV or using a computer, tablet computer or smartphone) • A range of 10–13 h of good quality sleep, including naps, with regular sleep and wake-up times

Dissemination has been defined as an active and planned process through which information is distributed to a target audience (16). In relation to the movement behaviors of children, parents and teachers are known to be relevant agents of change (17, 18). Thus, deliberate and strategic dissemination of the guidelines, with parents and teachers as the audience, is warranted. Stakeholder engagement is essential to facilitate effective dissemination and uptake of the guidelines. In related work in other countries (i.e., Canada, Australia), stakeholders consist of expert representatives from the education and health sectors (e.g., ECE administrators, health researchers), practitioners (i.e., teachers, principals), and parents (19, 20). Stakeholders' perceptions comprise one of the most frequently cited components when disseminating information to target audiences (16). In South Africa, where similar movement guidelines have been launched, the evidence suggests that stakeholders' perceptions helped assess the acceptability of the recommendations to the local population (21). Qualitative studies have revealed that stakeholders generally found their country-specific movement guidelines acceptable and aligned with health and education priorities (19–21). Barriers to the uptake of the guidelines have also been identified, such as lack of available information, daily activities that compete for time and resources, challenges associated with current social norms (19), and high usage of technology-based devices at home (20). The analysis of stakeholders' needs can also enhance the likelihood of the guidelines' uptake and identify strategies that can facilitate implementation. For instance, it has been suggested that ECE settings provide a natural avenue for dissemination, where multiple communication channels and a combination of media formats would reach parents and teachers. While findings from previous research can inform the dissemination strategies of the movement guidelines for young children in Hong Kong, formative context-specific work is imperative. Despite the availability of evidence from other countries, we cannot assume that similar approaches will be suitable and effective. From an implementation science perspective, health promotion strategies can be successful if local contexts and nuances are analyzed and stakeholders' needs are addressed (22).

In the current project, we aimed to generate evidence-informed insights for the design of dissemination strategies for teachers and parents of children in ECE. We sought to answer the following research questions: (a) *What are stakeholders' awareness and knowledge levels of the movement guidelines for young children in Hong Kong?* and (b) *What are the factors that influence the uptake of the movement guidelines, which should be considered in designing dissemination strategies?* By answering the first research question, we will be able to assess the starting point for the local end users of the guidelines. The second question, on the other hand, will help us leverage the available strengths and mitigate weaknesses in the current systems. Using a mixed-methods study design, we sought to (a) assess the stakeholders' levels of awareness and knowledge of the Hong Kong movement guidelines for young children and (b) obtain the insights of key informants, ECE teachers, and parents of children aged 2–6 years on the factors that influence the guidelines' uptake. In addition, our initial engagements with the key informants and stakeholders indicated that we needed to engage with domestic workers who contribute to the dynamics of caring for young children in Hong Kong.

## Materials and methods

Previous studies of stakeholders' insights have used qualitative designs where interviews revealed individual views, and focus groups explored shared experiences effectively. We adopted a mixed-methods triangulation convergence model to utilize the complementary strengths of quantitative and qualitative methods and gain a comprehensive view of the stakeholders' needs (23). Data were gathered through (a) an online survey of ECE teachers, (b) focus group discussions involving ECE teachers and parents of young children, and (c) individual interviews of key informants and domestic workers who cared for children. We collected data between December 2020 and March 2021, when schools and ECE centers were suspended in Hong Kong due to the pandemic. All procedures were reviewed and approved by the research ethics committee of the first author's affiliated university (Reference number 2019-2020-0145).

### Survey

Invitations were emailed to all registered local ECE centers across the Hong Kong territory. All ECE teachers were eligible to respond to an online survey hosted on Qualtrics. Valid responses were received from 314 ECE teachers from the three regions of Hong Kong (i.e., Hong Kong Island, Kowloon, and the New Territories). *Post hoc* calculation showed that the sample size was adequate to keep the margin of error at 4.6% with a confidence interval of 90%, given that the total population of ECE teachers at that time was 13,486 (24). The respondents consisted of 90.0% females. The majority of them were aged between 25 and 34 years (42.4%) or 35 and 44 years (30.3%); the rest were aged between 18 and 24 (10.5%), 45 and 54 (11.5%), and 55 and 60 (4.5%) years. Their mean years of experience was 12.4 years (SD = 9.32). Most respondents (58.0%) had a bachelor's degree qualification; relatively fewer had a sub-degree diploma (28.7%) or a postgraduate degree (13.4%). The participants' demographics are consistent with those of the ECE teaching workforce in Hong Kong.

The survey consisted of close-ended dichotomous questions (i.e., answered by Yes or No) that were adapted from previous studies to determine the teachers' awareness (e.g., “*Have you seen, heard, or read anything about guidelines for young children in physical activity?”*) and knowledge (e.g., “*Do you know the recommended minimum amount (in minutes) of physical activity per day for children aged 3–6 years?”*) of physical activity, sedentary behavior, and sleep guidelines (25, 26). An open-ended follow-up asked those participants who claimed to know the guidelines to indicate the minutes or hours recommended for each of the movement behaviors (e.g., “*Yes, I know the recommendation, it is…”*). Responses to the open-ended follow-up that matched the CHP guidelines were categorized as correct knowledge. Further additional questions enquired about the contexts in which the participants promoted the movement guidelines in ECE settings.

### Focus groups

From the list of registered local ECE centers, we invited six centers that represented the three Hong Kong regions to participate in the focus groups (i.e., two each from each region). We conducted twelve focus groups involving teachers (*n* = 18) and parents (*n* = 18) of children enrolled in the respective centers. One focus group was conducted separately for groups of teachers or parents in each ECE center. All teachers with at least 1 year of experience in the participating kindergarten were eligible to participate in the focus groups. However, we purposively formed groups where teachers taught at the nursery, junior kindergarten, and senior kindergarten levels to obtain diverse representations. Similarly, the invitation was open to all parents of children enrolled in the kindergartens. However, we formed the groups to have representation from parents of children in the three levels as aforementioned.

The teachers were all females, and they had been in their current roles for between 2 and 8 years. The parents were mostly mothers (88.8%). Two experienced researchers facilitated the discussions in the participants' first language (i.e., Cantonese), which explored the participants' (a) *awareness and knowledge of the movement guidelines for young children in Hong Kong* and (b) *perspectives on the factors that impact the uptake of these guidelines*. Open-ended questions were used to initiate the discussions with participants (e.g., “*What makes it difficult for teachers to help reduce sedentary behaviors among pupils?*”). To mitigate the possibility that some participants would dominate the discussion, the facilitators prompted each participant to contribute their insights before transitioning to the next topic (27).

### Interviews

Individual interviews were conducted with key informants (*n* = 7) who were purposively recruited to represent the knowledge and practice areas relevant to young children's health behaviors (19–21). The following participants were deemed knowledgeable of the local context: ECE principal (for children aged 3–6 years), nursery program director (for children aged 2 years and below), education leadership and management consultant, family psychologist, physical fitness specialist, early childhood education academic, and public health academic. The participants had been in their current roles for more than 6 years and had been in their respective industries (i.e., education, counseling, health and fitness) for more than 10 years.

As all the key informants performed their professional roles in bilingual (i.e., English and Chinese) environments, the interviews were conducted in English. Two experienced researchers explored the participants' (a) *understanding of the system factors that affect the movement behaviors of young children in Hong Kong* and (b) *perspectives on how the movement guidelines could be understood and taken up by teachers and parents in the local ECE context*. The open-ended questions were exploratory and sought to obtain participants' insights based on their own experiences in their respective roles (e.g., “*Based on your experiences with Hong Kong families, what are the factors in the local context that influence the physical activity participation of young children?” and “What do you think are the key considerations if we want parents to be motivated to help their children meet the movement guidelines?*”).

Teachers, parents, and key informants mentioned the roles of domestic workers in children's movement behaviors. The researchers, therefore, deemed it valuable to seek their insights. Further individual interviews were conducted with a convenience sample of domestic workers who provided childcare for young children (*n* = 7). In Hong Kong, middle-class families commonly employ a domestic worker as a form of alternative childcare when parents are both working (28). The domestic worker participants were all females and had been providing care for children in Hong Kong in the last 2–6 years.

Two experienced researchers conducted the interviews in the participants' first language (i.e., Tagalog). Open-ended questions explored the participants' (a) *awareness and knowledge of the movement guidelines for young children* and (b) *caregiving roles related to physical activity, sedentary screen time, and sleep of children* (e.g., “*In your daily routines with the child that you are caring for, does he/she engage in physical activities?”, “To what extent are you able to regulate the use of screens/gadgets by the child you are caring for?*”).

### Data analysis

The data gathered from the survey were analyzed using descriptive statistics to determine the percentages of teachers who were aware and knowledgeable of the movement guidelines. The relationships of teachers' characteristics with their awareness and knowledge of the movement guidelines were examined using the Chi-square test (for categorical data) and Spearman's rank correlation coefficient (for ordinal data). Statistical significance was set at *p* < 0.05, and all analyses were conducted using IBM SPSS 27.

The data from the focus groups and interviews were transcribed verbatim by researchers who had native proficiency in the language used (i.e., Cantonese, English, Tagalog). The Cantonese and Tagalog transcripts were subsequently translated into English by bilingual researchers. To preserve confidentiality, codes were assigned to each participant according to their group (i.e., T – teacher, P – parent, I – key informant, W – domestic worker) and number (e.g., T01 – teacher, participant 1). These codes are used when quotes are presented in this paper.

A realist framework for qualitative analysis was adopted in which language is assumed to capture participants' experiences of reality (29). Thematic analysis was conducted following a six-phase analytic approach: familiarizing with the data, generating codes, generating initial themes, reviewing and developing themes, refining, defining, and naming themes, and writing the report (30). The research questions of this study (i.e., awareness, knowledge, and factors that influence the uptake of the movement guidelines) directed the focus areas for the analysis. In examining the factors, coding and theme generation were inductive (i.e., bottom up) and semantic (i.e., the explicit meaning of gathered data) to align with the realist framework (29). To ensure the trustworthiness of the analysis, we took a team approach throughout the analytic phases. A team of three members (i.e., the principal investigator and two bilingual researchers each for English/Cantonese and English/Tagalog) read the transcripts independently, where the bilingual members read both the original and translated transcripts to ensure accuracy. Three rounds of iterative discussions were conducted to generate codes and develop themes. The discussions deliberately explored multiple interpretations and reflexivity (31). The interpretations were subsequently discussed with the wider research team as the themes were reviewed and refined.

## Findings

### Stakeholders' awareness and knowledge of the movement guidelines

The findings from the online survey are summarized in Table 2. The majority of the teachers reported being aware of the guidelines for physical activity (86.9%), sedentary screen time (70.1%), and sleep (58.3%). Most teachers also reported that they knew the recommended minimum time for physical activity (85.4%), the maximum time for using screens/gadgets (79.9%), and the minimum amount of sleep (83.8%) for young children. Based on the open-ended question, it was determined that smaller portions of the teachers reported the correct recommended time for physical activity (19.4%), sedentary behavior (18.2%), and sleep (36.6%).

**Table 2:** Percentage of teachers (*N* = 314) who are aware and knowledgeable of the movement guidelines.

	**Movement guidelines**		
	**Physical activity**	**Sedentary behavior**	**Sleep**
**Awareness of the guidelines**
Aware	86.9	70.1	58.3
Not sure	9.9	23.6	29.6
Not aware	3.2	6.3	12.1
**Reported knowledge** ^a^
Knowledgeable	85.4	79.9	83.8
No idea	14.6	20.1	16.2
**Actual knowledge** ^b^
Correct knowledge	19.4	18.2	36.6
Incorrect knowledge	66.0	61.7	47.2
No idea	14.6	20.1	16.2

Data are presented as percentages.

a The respondents reported whether they knew the specifications of the movement guidelines or they had no idea.

b The respondents indicated the specifications of the movement guidelines.

Teachers with higher degree qualifications tended to be more aware of the guidelines for physical activity (χ^2^ = 11.110, *p* = 0.025) and sedentary screen time (χ^2^ = 15.130, *p* = 0.004) compared to those with lower qualifications. Those who had more years of experience were also more likely to be aware of the guidelines for physical activity (*r* = 0.171, *p* = 0.002) and sedentary screen time (*r* = 0.120, *p* = 0.033). Neither qualification nor experience was associated with knowledge of the movement guidelines. The frequently reported contexts in which physical activity was promoted in ECE settings included free play periods (86.0%), outdoor activities (77.1%), indoor games (68.8%), and integration with learning areas such as literacy and numeracy (30.9%).

The majority of the teachers who participated in the focus groups reported that they were aware of the movement guidelines (83.3%). All the teachers agreed that the guidelines were suitable for young children in Hong Kong because they were evidence-based. In terms of meeting the guidelines, all the teachers believed that no more than half of their pupils typically met the daily physical activity guidelines. Furthermore, they believed that most pupils typically exceed the limits for sedentary screen time and do not have adequate sleep.

A small portion of the parents (17.7%) who participated in the focus groups were aware of the movement guidelines for young children. When presented with the specific recommendations for physical activity, sedentary behavior, and sleep, they all agreed that they were suitable for their children. However, the discussion revealed that a minority of them reported that their children could meet the physical activity guidelines (33.3%). There appeared to be relatively more parents who reported that their children could meet the sedentary screen time limits (66.7%) and accumulate adequate sleep (61.1%).

Only one domestic worker was aware and had knowledge of the movement guidelines for young children. While most of them expressed ideas of how much physical activity, sedentary screen time, and sleep are ideal for young children, those ideas did not match the guidelines. When presented with the correct information of the movement guidelines, none of the domestic workers had an opinion on their suitability for the children. Instead, all of them pointed to the parents as those who may judge whether such guidelines are suitable for their children. Only one domestic worker shared routines where the child appeared to meet the movement guidelines; the rest of them described routines where the children appeared to have insufficient physical activity, excessive sedentary screen time, and inadequate sleep. When describing their childcare roles in relation to the movement guidelines, the domestic workers discussed looking after children during outdoor activities and taking them to and from school. Regarding the use of digital devices and sleep, the domestic workers expressed that they generally followed the instructions of the parents.

### Enablers: Factors that support the uptake of the movement guidelines

Drawn from the responses of teachers, parents, and key informants, four enablers were identified in relation to promoting the uptake of movement guidelines for young children. These are summarized in Table 3, along with the subthemes and sample quotes.

**Table 3:** Factors that enable children's compliance with movement guidelines.

**Theme**	**Subthemes/description**	**Example quotes**
Parent engagement	*Parents' knowledge* of the movement guidelines, understanding of the rationale (“*why”*), and knowledge of strategies to implement the guidelines at home (“*how”*)	“If parents know that if their children do physical activity, their development will be affected positively… it helps us reach our goal.” (T03) “Parents need to understand that physical and cognitive development are equally important.” (I02)
	*Parenting skills* that facilitate children toward meeting the guidelines. These include parents' policies at home to set routines (e.g., sleeping time) or regulate activities (e.g., screen time) of children. Includes the consistency between parents' knowledge and what is implemented at home	“Some parents are stricter – they may not truly allow their children to use devices. However, some cannot control the emotions of the children, so they give the devices when they are acting up.” (T15) “There are parents of that don't let their children watch TV too much. They are usually able to limit the time within an hour.” (T12)
	*Accessible support* for parents underpins parent engagement	“Let parents know what they can do – recommend them some games to play… find some community resources or exercise video for them to do at home” (T09) “What can we do within such a small space at home? Maybe you can provide more activity suggestions with different materials that we can find at home?” (P03)
Role of ECE centers	The *curriculum content* in ECE centers and learning activities that enhance physical activity participation, reduce sedentary screen time, and promote healthy sleep patterns	“In fact we [parents] lack systematic knowledge to implement WHO standards… schools can systematically include [them] into the syllabus” (P13) “Teachers can appreciate that it does not have to be physical education… instead, it has to be integrated with lessons and routines” (I06)
	*Teachers' adequate knowledge and skills* to implement suitable activities	“If health experts would train teachers, they [teachers] would in turn know to manage movement guidelines, and help parents in promoting them at home” (I02) “Children will to listen to the teacher' words instead of parents'. When the teacher knows the guide and says we cannot watch screen for longer than the guide, it is better.”(P015)
	*Accessible support* for ECE centers and teachers	“The teachers can be supported in designing materials and implementing strategies to help children meet these guidelines.” (I04) “We need support so that we can discuss with the curriculum team, to manage the many conflicts in the current. I think it is progressing gradually. There is still a lot of content that tends to be rushed.” (T16)
Home-school cooperation	A *two-way relationship between teachers and parents*, such that activities in the ECE center may be followed up at home; this requires that both teachers and parents have the knowledge and understanding of the movement guidelines	“Parents and children can exercise together, afterwards they can be more energetic together. We [teachers] can provide some advice to see if their activities work, and we can work with them to improve activities.”(T05) “Some parents may not have the habit of physical activity themselves, or do not understand the guidelines… schools can help share knowledge” (I03)
Community activities	The *availability of activities that can be considered forms of physical activity* in the community outside of school, which young children can join; this requires a mechanism for such information about activities to reach parents	“A civic group held a parent–child hiking activity and social workers told us which trails are suitable for kids to hike” (P11) “I can't think of more activities, so I search for classes in the community center” (P05)

#### Teachers

From the teachers' discussions, two themes were identified as enablers: (a) *parents' engagement* and (b) *availability of community activities*. The teachers described parents' engagement in relation to their knowledge and understanding of the movement guidelines, including the impact of physical activity, sedentary behaviors, and sleep on the overall health and development of young children. They further noted that when parents have a good understanding of the “*what”* and “*why”* underlying the guidelines, they are more likely to help their children meet them. Parents' engagement was also related to their ability to do physical activities with their children at home, regulate the use of digital devices, and implement regular sleep routines. The parents also need to have a sense of “*how”* to implement the movement guidelines. Practical information can give parents clear ideas on how children can avoid excessive sedentary screen time and accrue adequate physical activity and sleep. The availability of activities in the community was discussed concerning practical ideas that parents can use. Specifically, parents could register their children for community activities available in their locale (e.g., sports programs, dance lessons) if they were aware that such programs existed.

The teachers noted that parents' engagement is crucially linked to parenting skills. They determine where routines are established, children's timetables are managed, and desired behaviors that meet the movement guidelines are modeled.

#### Parents

From the parents' discussions, two themes were found to be consistent with those shared by the teachers: (a) *availability of community activities* and (b) *support for parents' engagement*. Similar to the teachers, the parents also expressed the importance of community activities. The parents appeared to rely on community activities when they needed childcare support –, i.e., when children are attending community activities such as swimming lessons, someone is looking after them. The parents also described community activities as options for family activities.

In terms of parents' engagement, the participants expressed that they could promote healthy movement behaviors when they had support. The parents need updated knowledge of what is beneficial and what is harmful in relation to physical activities, sedentary behaviors, and sleep. They described some common beliefs that appear to be inaccurate, such as children not needing to exercise. It also appeared that prompts to seek specific information about the movement guidelines are important because parents do not know what to look for otherwise. The parents also expressed that they could promote physical activity if they had practical ideas. Support in relation to managing sedentary screen time was frequently mentioned and appeared to be the most critical.

Two other enabling factors were identified in the parents' discussions: (a) *roles played by the ECE teachers* and (b) *opportunities for children to socialize*. The parents believed that ECE teachers have knowledge about movement behaviors and are able to integrate them into their teaching. It was also noted that teachers could explicitly teach their pupils about healthy movement behaviors, which the parents expected to have a greater influence on the children. The parents also recognized that opportunities to socialize with other children are an important enabler because they promote consistent engagement. The presence of playmates in outdoor settings (e.g., playground, park) tends to make children engage better. Siblings also appeared to encourage physical activity participation.

#### Key informants

From the key informants, *support for parents' engagement* was also identified as an enabling factor. While their responses were consistent with the parents' discussions, the key informants highlighted that in addition to strengthening parents' knowledge, facilitating parents' motivation to promote the recommended movement behaviors is needed. To enhance motivation, supporting programs can counter parents' beliefs that physical activities and play are not as important as academic pursuits. Drawing from their experiences with local families, the key informants noted that support for parents includes acknowledging the constraints faced by the working class. Workshops may help improve parents' knowledge, but support should be consistent.

The key informants identified two other enabling factors: (a) *home-school cooperation* and (b) *support for ECE teachers in their roles*. Cooperation between the parents and teachers was deemed essential for promoting the uptake of the movement guidelines. The key informants from the education sector acknowledged the packed ECE curriculum. Teachers consistently try to meet government-mandated requirements, which tends to limit their capacity to integrate movement behaviors into their curricula. Activities across a continuum from school to home, therefore, are crucial to improve the uptake of movement guidelines. Moreover, the key informants believed that teachers are effective messengers who can share knowledge with parents and encourage changes in mindset and routines.

As noted above, parents also identified the role of ECE teachers in enabling the uptake of the movement guidelines. For the key informants, however, the support provided to the teachers so they can deliver the role of enhancing parents' awareness and knowledge is a key enabler. As ECE teachers need to design classroom activities within the constraints of curricular demands, it is crucial that principals buy into the value of the movement guidelines because they have the authority to endorse curricular modifications. Thus, one form of support is targeted messaging for principals. The key informants further described support in the form of on-site and bespoke workshops that will enhance the teachers' ability to design classroom and home-based activities that are integrated into learning areas and daily routines.

### Challenges: Factors that hinder uptake of the movement guidelines

Five challenges were identified from the participants' responses. The subthemes and sample quotes are summarized in Table 4.

**Table 4:** Factors that challenge children's compliance with movement guidelines.

**Theme**	**Subtheme/description**	**Example quotes**
Time poverty of parents	*Parents' lack time* to engage with their children to promote the movement guidelines, due to demanding work hours	“Most of us are both working parents and there is a problem of who will do activities with the children” (P02) “If parents take the mobile phone away, the children get upset. Therefore, parents do not take the mobile phone away when they need to go out to work” (T04)
	*Parents experience fatigue* and lack of energy which affects their engagement with their children's movement behaviors	“Most parents want to rest, and so don't want to exercise. As a result, their kids will not exercise too” (T11) “Parents are already exhausted with work to follow through with the guidelines” (I01)
Curriculum requirements	*Traditional learning areas* (e.g., literacy, numeracy) of the ECE curriculum, altogether tend to fill up the allocated ECE class hours	“Kids have lots of things to learn like English so they may not have too much time to play” (P06) “There is very little room left for the ECE curriculum to involve physical activity” (I04)
	Significant amounts of *homework* are typically given to children in ECE	“There is a lot of homework, and parents want to see their children complete their academic work. Therefore, the playing time in the playground becomes limited” (T02). “As the kids have much homework, after they finish and do the revision, it's already late to go out and exercise. It is difficult to do it.” (P08)
	*Teachers' perceive* that the time for conducting movement activities is insufficient	“We [teachers] know that the children will be happier if they play. However, it is hard to practice. The Education Bureau advises us, and we need to meet parents' expectations. Children need to learn the academics. In this perspective, there is no more time to move and play.” (T03) “Teachers focus on the academics and don't get the opportunities to explore and be better” (I05).
Limited physical space	There is limited space and resources in *ECE classrooms*	“The physical environment of the center is not big, so activities are limited” (T07) “Actually, it is not only the indoor place being too small; but it is an equipment problem. For example, the materials used in this class are not suitable to use in another class.” (T16)
	Average *Hong Kong homes* tend to have small floor area	“There is not enough space, but we can't take them out to play on weekdays because we don't have time.” (P07) “It is not that children do not want to exercise, it is because parents don't have time to play with them and there is not enough space at home. It is easy for children to play for 2–3 hours if there is space and time for them.” (P09)
	There is inconsistent availability and accessibility of *community spaces* (e.g., playgrounds, parks)	“It is difficult for ECE schools to book sports grounds; it is easier for primary and secondary schools” (T09). “Other places now have more parks for children but this is not the same for all places; there are more spaces dedicated to shopping malls.” (P08)
Social values	The society believes that *academic achievement is most important* and that playing outdoors may have inherentassociated risks (e.g., injuries, insect bites)	“Hong Kong people don't like sports.” (P11) “Play should be an adventure, but they [parents, teachers] always put safety as the first priority (I02)
Pandemic-related restrictions	The restrictions include *social distancing measures* and school suspensions, limiting the spaces that children can use	“Parents said that with the pandemic, they cannot bring their kids to the park as they thought it is risky” (T07) “We had the rope skipping activity. The problem is that due to the pandemic, we can't implement the activity. It is difficult for the children to breathe when they are wearing a mask. (P13)
	*There is a diminished contribution of the ECE centers* in children's accrual of physical activity	“Before the pandemic, we [ECE] would provide some physical activity time for children and they may ride a bike or walk to school” (T14). “Before the pandemic, I mostly rely on the school for exercise” (P17)

#### Teachers

The teachers' discussions revealed four themes as challenges to children meeting the movement guidelines: (a) *curriculum requirements that lead to time constraints*, (b) *limited space*, (c) *time poverty of parents*, and (d) *restrictions due to the COVID-19 pandemic*. The most widely discussed challenge was related to the local curriculum requirements. It was noted that the academic learning areas (e.g., numeracy, writing) tend to fill up the 3-h daily classes. Thus, the teachers find it difficult to find the time to implement physical activities. As parents are aware of curricular requirements, they also expect homework to be academically oriented. Thus, time at home tends to be focused on academics as well. It was also noted that recent changes in local regulations meant there is no more option for nap time for the typical half-day program.

The teachers discussed that ECE centers mostly have limited space, which presented a challenge to implementing physical activities. They seek options outside the center (e.g., community sports grounds), but they are typically less prioritized and struggle with booking. As such, activities tend to be mostly indoors, which teachers find challenging in relation to sustaining the children's interests. The teachers also noted that space issues are also present at home; hence, they need to support parents by giving them activities that can be done in small spaces.

With regard to the home situations, the teachers noted that parents typically do not have enough time to engage their children to meet the movement guidelines. They observed that parents' working hours affect children's routines, especially in relation to sleep – children sleep late because parents get home late from work. Moreover, the teachers believed that parents have difficulty regulating screen time because they prefer that their children not be upset by restrictions. Long working hours also mean that parents tend to be exhausted and have no energy to engage in physical activities with their children. Working parents manage the time issues by relying on the grandparents or domestic workers to look after the children, but the teachers noted that such arrangements often lead to children spending more time indoors and being sedentary. Thus, even if parents were to gain more knowledge about the movement guidelines, implementing them may remain a challenge as parents attempt to manage their time by relying on the grandparents or domestic workers.

The last challenge identified by the teachers relates to the restrictions due to the COVID-19 pandemic. The most frequently mentioned issue was the suspension of schools, and the teachers noted the impact on physical activities. From 2020 to 2021, schools across all levels in Hong Kong had suspended periods during which time children were mostly confined to their homes. The teachers observed that initially, many parents were concerned of infection and thus kept their children indoors. Playgrounds and public spaces were closed such that even when parents allowed their children to go out, the spaces remained restricted. Finally, despite curricular limitations, the teachers thought that children were able to accrue greater physical activity when they attended school compared to the periods of school suspension.

#### Parents

The following challenges were identified from the parents' discussions: (a) *time poverty*, (b) *limited facilities and space*, (c) *restrictions due to the COVID-19 pandemic* and (d) *values held by Hong Kong society*. The parents confirmed that they struggle with finding time to help their children meet the movement guidelines. This is especially true for those families where both parents are working. Due to limited time, grandparents and domestic workers assume childcare roles, which the parents acknowledged are not conducive to children meeting the movement guidelines. Families where one parent is full-time at home appear to be more capable of promoting the movement guidelines because at least one parent is able to implement routines firmly.

The parents also discussed the challenges associated with limited spaces at home and in the community. Combined with parents' time poverty, the typically small homes in Hong Kong mean that parents can promote physical activities only on days that they are off work and they can go out with their children. Space limitation also appears to be related to parents' lack of knowledge because they reported not knowing what to do inside small home spaces. The parents appeared to rely on shared community spaces as venues for physical activity (i.e., public playgrounds, parks, rooftop areas). However, they deemed these community spaces inadequate and inconsistent. Many facilities also charge fees, which limits the accessibility for working class families.

For the parents, the main issue with the pandemic-related restrictions was that children were not able to go out either to go to school or to play outdoors. Parents discussed that without school activities, their children tended to be inactive, and the challenges associated with small home spaces were aggravated. Some parents discussed attempts to do structured activities with their children, but these were infrequent and perceived as an additional burden. Even after the restrictions were lifted and children were able to use community spaces, the parents noticed that socialization opportunities did not truly resume.

Finally, the parents identified challenges associated with the values upheld by Hong Kong society. The most discussed point is the importance of academic achievement, which encourages parents to focus on academic learning beginning from ECE. Despite focusing on academics themselves, most of the parents appear to be aware that it is not helpful with the movement guidelines. Some parents also noted that physical activity and sports are not generally valued in Hong Kong, which they felt makes it difficult to find opportunities for their children to join. Social values also contribute to grandparents who are not supportive of letting children engage in physical play.

#### Key informants

Similar to the challenges identified by the teachers and parents, the key informants identified the following: (a) *time poverty*, (b) *curriculum requirements*, and (c) *values held by Hong Kong society*. The challenge associated with working parents' limited time came up once again, where key informants from the education sector noted that working parents could not actively promote the movement guidelines even if they had knowledge of them. In particular, it was noted that work demands leave the parents exhausted.

Key informants from the health and family sector also noted that there is very little room for promoting movement behaviors in the current curriculum. Consequently, teachers need to focus on meeting academic expectations and eventually have little practice with promoting physical activities. Finally, the prevailing values of Hong Kong society were related to high levels of concern for safety. Informants from both the education and health sectors noted that safety is always prioritized by both parents and teachers, limiting the opportunities for physical play.

## Discussion

The majority of ECE teachers in Hong Kong appear to have high awareness of the movement guidelines for young children, especially those with higher degree qualifications and longer working experience. Most of the teachers also claimed to know the guidelines, but those who had the correct knowledge were much fewer. Parents, on the other hand, had limited awareness and knowledge of the movement guidelines. The domestic workers, who represent an extension of childcare at home, also reported limited awareness and knowledge of the movement guidelines. These findings suggest that dissemination strategies are crucially needed so that adults who may influence children's movement behaviors are aware of and have the correct knowledge of evidence-based recommendations.

In Canada, Australia, and South Africa, where similar studies informed the dissemination of movement guidelines for children, the stakeholders mainly included teachers and parents (19–21). The Hong Kong context introduced the roles of additional carers (i.e., grandparents and domestic workers), comparable to other Asian cities and countries. Nevertheless, the primary targets of our dissemination strategy remain ECE teachers and parents, as they appear to have control over policies and activities in classrooms and at home. They have also been known to be relevant agents that can influence the movement behaviors of children (17, 18). While domestic workers look after the children during the parents' working hours, our findings suggest that they do not have autonomy in how they deliver their roles in relation to the movement behaviors of children. There may be benefits to dissemination strategies for domestic workers, but such efforts should be through the parents given the dependency of domestic workers on the parents' views. For example, we can provide parents with materials that they could readily share with domestic workers and grandparents (e.g., activity cards, YouTube videos).

There was a consensus across stakeholders and key informants that the movement guidelines adopted by the CHP are appropriate and suitable for young children in Hong Kong. The movement guidelines were similarly found acceptable by local stakeholders in other countries (19–21). Despite the positive local regard for the guidelines, reports from the stakeholders suggest that most children do not meet them. These findings are consistent with the Department of Health reporting that children in ECE are not sufficiently active and spend more than 1 h per day on their devices (4).

### Design of dissemination strategies

The factors that enable and challenge the uptake of the movement guidelines among young children in Hong Kong appear to cut across local (i.e., home, ECE center) and system contexts (i.e., curriculum policies, social expectations). Time is the most critical factor, which seems to impact movement behaviors at home and in ECE centers. In Canada, time was also an issue, as other daily activities were perceived to compete with physical activities (19). In the case of Hong Kong, our findings suggest that the time issue is more significant where there seems to be time poverty, which can be defined as having scarce discretionary time (i.e., outside of work) that may be allocated to health-related behaviors (32). Time poverty has been associated with poor wellbeing and physical health, and it has been argued that policies need to address this issue at the systemic level (33). It has been identified as a critical barrier to an individual's physical activity participation (34). In this study, we found that parents' time poverty manifests as a similar barrier to their children meeting the movement guidelines. Parental time was particularly highlighted among working-class families. For the ECE centers, time issues are related to the policies of the local education system, which affect how teachers allocate class time. ECE teachers in Hong Kong have noted that even the primary school curriculum has an impact on their priorities (35) because they need to ensure that children transition well from senior kindergarten.

Current social norms have also challenged the uptake of movement guidelines (19). With the prevailing social values highlighted in this current study, movement behaviors tend to be less prioritized in ECE centers. Beliefs and attitudes of the wider society cannot be changed in the short term; hence, dissemination of the movement guidelines will have to work around recognizing that academic pursuits will likely remain a priority.

Considering that dissemination is an active and deliberate process of distributing information to specific target audiences (16), the identified enablers and challenges to the uptake of movement guidelines can inform the design of dissemination strategies. For teachers, we need to include components that will help them promote physical activities without taking time away from the academic learning areas. To this end, teachers can be supported in designing learning activities where movement is integrated with academics (e.g., mathematics, language). It has been suggested that integrated activities have the potential to help children meet physical activity guidelines (36, 37). In recent work in Hong Kong, integrated gross motor skills and mathematics successfully increased the physical activity accrual of young children without any detrimental effect on mathematics abilities (38). Leveraging home-school cooperation, teachers can also design physically active homework for children.

Dissemination strategies for parents need to acknowledge the realities of working families and the impact of this on time. Like income, time is a finite resource that parents need to allocate among competing demands (39). It is therefore necessary to help parents access a range of practical ideas for them to facilitate short bouts of physical activity at home. Online resources for physical activity, which had grown exponentially during the pandemic (40), can be curated and shared with parents (e.g., dancing or yoga can be done by children with little supervision required of parents). The use of such online resources also shifts the nature of screen time from sedentary to active.

The challenge of space limitation, which cuts across homes, schools, and community public spaces, is not surprising. High-rise housing is dominant in Hong Kong, where middle-class families live in relatively compact and small apartments (41) and public spaces tend to be small in mass housing areas compared to upmarket and commercial-business zones (42). Schools also have limited physical spaces, many of which have little opportunity to expand (43). In this context, teachers and parents tend to think that the lack of space prevents physical activities. However, physical activities can be taken up even in small spaces (44). Dissemination strategies might show teachers and parents that activities can be designed for small spaces, including movements within compact households.

Not surprisingly, the impact of the COVID-19 pandemic was highly discussed by teachers and parents. Recent studies have shown decreased physical activity, increased sedentary behaviors, and disrupted sleep in children during the pandemic (45, 46). The main restriction was related to school suspension, where parents struggled to promote movement behaviors, especially physical activity. In a post-lockdown context, our findings highlight the role of the ECE sector in promoting movement guidelines. As ECE teachers are key contributors to enhancing young children's activity levels (47), dissemination strategies need to prepare them to intentionally provide opportunities that promote the uptake of movement guidelines.

Bringing together the findings of this study, we recommend that in contexts where there is pervasive time poverty, space limitations, or high priority for academic achievements, dissemination strategies for the movement guidelines of young children should consider the following:

- Ensuring that not only awareness but also the correct knowledge of the guidelines is promoted among the adults that influence the movement behaviors of young children (i.e., teachers, parents, domestic workers, grandparents);
- Equipping teachers with the ability to design and deliver intentional activities that integrate movement with academic learning areas and that can be implemented in relatively small spaces;
- Directing parents to curated and publicly available resources that they can access for small-space home physical activities, managing sedentary screen time and sleep, and community activities that their children can join; such activities need to be viable despite parents having limited time after work;
- Enabling the ECE centers to include practices that promote movement guidelines as they cooperate with the parents and share information in their wider communities.

### Strengths and limitations

Taking a broader approach to examining stakeholders' views in relation to the movement guidelines for children, we adopted a mixed-methods design. This allowed us to assess the teachers' awareness and knowledge levels across Hong Kong, thus setting us up for future monitoring. However, we acknowledge that our survey respondents make up a convenience sample, which may limit our generalizations. We did not perform a similar survey with parents, and future work is needed to enable a monitoring strategy. While we engaged with domestic workers, their insights were relatively limited, and future exploratory work may be of value. Grandparents were identified as having roles in caring for children, but we did not engage with them in this study. Further work is needed to explore their decision-making roles in relation to parents and the movement behaviors of young children.

While our findings are specifically drawn from the Hong Kong context, the enablers and challenges that we identified may be present in places where comparable circumstances exist (e.g., Macau, Singapore). In places where education policies place time constraints on ECE curricula or the socio-economic environment leads to parents' time poverty, dissemination of movement guidelines for children may consider the strategies identified through this research.

Our study was implemented during the COVID-19 pandemic. We gained insights that helped us better understand the movement behaviors of children in these unprecedented social circumstances. On the other hand, we also need to consider that the enablers and challenges that we identified may be in flux as we move forward toward a post-pandemic world. Further study is needed to evaluate the impact of the dissemination strategies proposed in this current work. For instance, an implementation science approach can be adopted to evaluate outcomes (22) while fully considering the pandemic-associated shifts in Hong Kong's health policies. Evidence from evaluations, can be utilized by government agencies in seeking long-term and sustainable promotion of healthy movement behaviors in young children. Finally, future research is needed to address the prevalent social values in Hong Kong – and in other East Asian societies – that are viewed as challenges to physical activity in early childhood.

## Conclusion

We found that in Hong Kong, teachers are aware of the movement guidelines for young children, but their knowledge of the specific guidelines is inaccurate. On the other hand, parents and domestic workers have limited awareness and knowledge levels. We recommend that dissemination strategies in ECE centers should not only deliver knowledge content but also enable stakeholders to mitigate the challenges associated with time and space limitations. The dissemination of movement guidelines for children in societies facing similar conditions may consider adopting the recommendations we presented. We acknowledge that dissemination is an ongoing process that needs to involve stakeholders and policy-makers, for which further research is needed. Finally, implementation science frameworks may be used in future work to assess dissemination strategies and determine immediate and long-term outcomes of children's physical wellbeing.

## Data availability statement

The raw data supporting the conclusions of this article will be made available by the authors, without undue reservation.

## Ethics statement

The studies involving human participants were reviewed and approved by Human Research and Ethics Committee, The Education University of Hong Kong. The patients/participants provided their written informed consent to participate in this study.

## Author contributions

CC is the principal investigator of the grant and is responsible for the overall concept, design, and implementation of the study. RJ, CN, CS, and KC are co-investigators of the grant who collaborated from proposal preparation to project implementation. CC drafted the manuscript, and all co-investigators provided expert input within their areas of expertise. All authors contributed to this paper and approved the submission.

## Funding

Health and Medical Research Fund, Food and Health Bureau, Government of Hong Kong (Reference no. 17180131).

## Conflict of interest

The authors declare that the research was conducted in the absence of any commercial or financial relationships that could be construed as a potential conflict of interest.

## Publisher's note

All claims expressed in this article are solely those of the authors and do not necessarily represent those of their affiliated organizations, or those of the publisher, the editors and the reviewers. Any product that may be evaluated in this article, or claim that may be made by its manufacturer, is not guaranteed or endorsed by the publisher.
